# Editorial: New insights in the microbe-vector interaction

**DOI:** 10.3389/fmicb.2024.1364989

**Published:** 2024-01-16

**Authors:** Yong Qi, Jinwei Zhang, Marcos Rogério André, Tian Qin

**Affiliations:** ^1^Huadong Research Institute for Medicine and Biotechniques, Nanjing, Jiangsu, China; ^2^Department of Anesthesiology, Nanjing Drum Tower Hospital, Affiliated Hospital of Medical School, Nanjing University, Nanjing, Jiangsu, China; ^3^Vector-Borne Bioagents Laboratory (VBBL), Department of Pathology, Reproduction and One Health, Faculty of Agricultural and Veterinary Sciences, São Paulo State University (UNESP), São Paulo, Brazil; ^4^National Key Laboratory of Intelligent Tracking and Forecasting for Infectious Diseases, National Institute for Communicable Disease Control and Prevention, Chinese Center for Disease Control and Prevention, Beijing, China

**Keywords:** vector, interaction, microbe, vectorborne diseases, pathogen

In recent years, there has been a growing awareness of emerging vector-borne diseases, leading to a substantial amount of research in this area. The vector-borne pathogens, although posing a potential health threat to various vertebrate hosts, including humans, appear to have little impact on their arthropod vectors, such as ticks, mosquitoes, fleas, sandflies, mites, etc. (Johnson, 2017). However, increasing knowledge suggests that these symbiotic microbes actually influence vector development, reproduction, metabolism, immunity, and competence (Wang et al., 2022b, 2023). Understanding these interactions between microbes and vectors is crucial for developing effective prevention and control strategies for vector-borne diseases, especially as translational applications aiming to use microbiota to develop non-chemical-based vector control approaches have emerged (Wang et al., 2022b, 2023). Therefore, the goal of this Research Topic is to gather the latest advances in our understanding of microbe-vector interactions and their role in the transmission of vector-borne diseases.

The interactions between microbes and vectors are believed to play a crucial role in the cross-species transmission of vector-borne pathogens. For instance, the ability of *Aedes* spp. mosquitoes to transmit multiple arboviruses involves a complex relationship among mosquitoes, their microbiome, and the viruses. In this Research Topic, Mantilla-Granados et al. have compiled a comprehensive review of the latest information about the arbovirus infection process in *Aedes* spp., the source of mosquito microbiota, and its interaction with the arbovirus infection process, in terms of its implications for vectorial competence. This review summarizes the arbovirus-causing innate immunological pathways and adaptive responses in mosquitoes and their mechanisms. It also analyzes the general sources of the *Aedes* mosquito microbiota, and their direct or indirect influences on vector competence, indicating the complexity of this relationship influenced by intrinsic and extrinsic conditions at different geographical scales. Manipulation of mosquito microbiota is believed to affect vectorial competence, representing a promising direction for developing strategies to control arbovirus transmission. However, the interactions between mosquitoes, arboviruses, and their associated microbiota are yet to be thoroughly investigated.

Pathogens typically colonize the midgut and salivary glands of vectors, making these organs prime targets for microbiota manipulations. In a study by Piloto-Sardi et al. the salivary gland and midgut microbiomes of the soft ticks *Ornithodoros erraticus* and *Ornithodoros moubata*, the main vectors of African swine fever virus and human relapsing fever spirochetes, were analyzed and compared. The study revealed different taxonomic structures of the bacterial microbiome in different organs of the same tick species, as well as in the same organs from different species. However, *Muribaculaceae* and *Alistipes* were identified as keystone taxa in the salivary glands shared by both tick species, suggesting their potential as candidates for anti-microbiota vaccines to alter the microbiome and impact tick physiology and/or pathogen colonization.

Various factors, including the host of vectors, influence the microbiota of the vectors, subsequently impacting pathogen transmission. In a study by Moore et al. in this Research Topic, various flea-borne pathogens were detected in the cat flea *Ctenocephalides felis* and their infesting cats, and the factors driving flea-borne pathogen presence and transmission were analyzed. This study emphasizes the importance of considering reservoir host attributes and vector phylogenetic diversity in epidemiological studies of vector-borne pathogens. Another study within our Research Topic characterizes the bacterial microbiome of non-hematophagous bats and their associated ectoparasites (including *Streblidae* flies and *Macronyssidae* and *Spinturnicidae* mites) in Brazil (Rogério André et al.). Medically significant bacteria were detected in both the samples of bats and their attaching ectoparasites. Importantly, this is the first time the bacterial community of bat-associated *Macronyssidae* and *Spinturnicidae* mites has been identified.

In recent times, numerous novel vector-borne pathogens have emerged, while old ones, such as the Ebola virus, are re-emerging or being discovered in non-traditional hosts (Soong and Dong, 2021; Zhou et al., 2022). This has significantly enhanced our understanding of microbe-vector interactions. In this Research Topic, Jin et al. identified 13 Rickettsiales species from the genera *Rickettsia, Anaplasma*, and *Ehrlichia*, including three putative species of *Ehrlichia* in five tick species. The findings reveal the extensive diversity of Rickettsiales bacteria in ticks in the investigated area and highlight a potential risk of infection for humans. Additionally, Kaewmee et al. detected *Leishmania* spp. in biting midges and newly identified *Culicoides peregrinus* as the natural vector responsible for the transmission of *Leishmania martiniquensis* in Thailand. They also identified and isolated novel *Crithidia* spp.

In addition, Wang et al. provide a summary of the most recent discoveries regarding the dynamic interaction between host autophagy and *Coxiella burnetii* infection, emphasizing the intricate strategies employed by the pathogen to manipulate its host autophagy and evade the host immune system. Studying the tactics used by pathogens to evade the immune systems of their vectors is crucial and a current focus of research, which may be inspired by this mini review.

Currently, cutting-edge tools such as metagenomic sequencing technology are accelerating the elucidation of microbe-vector interactions (Toranzos and Santiago-Rodriguez, 2022; Wang et al., 2022a). The comprehension of these interactions is growing. The application of microbe-vector interactions to control vector-borne diseases has become a focal point of research in the field and has demonstrated success in some areas (Wang et al., 2022a). However, due to the diverse life histories and habitats of vectors, the interaction between different vectors and their symbiotic microbes varies, and the interaction between the same vector and its symbiotic microbes can also change under different physiological states. Therefore, many aspects of the interactions between vectors and microbes, as well as their mechanisms, remain unknown and warrant further study (Song et al., 2022).

## Author contributions

YQ: Writing – original draft. JZ: Writing – review & editing. MA: Writing – review & editing. TQ: Writing – review & editing.

## References

[B1] Johnson N. (2017). Tick-virus interactions: toll sensing. Front. Cell. Inf. Microbiol. 7, 293. 10.3389/fcimb.2017.00293 28713778 PMC5492658

[B2] Song X. Zhong Z. Gao L. Weiss B. L. Wang J. (2022). Metabolic interactions between disease-transmitting vectors and their microbiota. Trends Parasitol. 38, 697–708. 10.1016/j.pt.2022.05.002 35643853

[B3] Soong L. Dong X. (2021). Emerging and re-emerging zoonoses are major and global challenges for public health. Zoonoses 1, 1. 10.15212/ZOONOSES-2021-0001

[B4] Toranzos G. A. Santiago-Rodriguez T. M. (2022). MULTI-OMICS as invaluable tools for the elucidation of host-microbe-microbiota interactions. Int. J. Mol. Sci. 23, 13303. 10.3390/ijms232113303 36362090 PMC9656217

[B5] Wang G. H. Du J. Chu C. Y. Madhav M. Hughes G. L. Champer J.. (2022a). Symbionts and gene drive: two strategies to combat vector-borne disease. Trends Genet. 38, 708–723. 10.1016/j.tig.2022.02.013 35314082

[B6] Wang J. Gao L. Aksoy S. (2023). Microbiota in disease-transmitting vectors. Nat. Rev. Microbiol. 21, 604–618. 10.1038/s41579-023-00901-6 37217793 PMC12397960

[B7] Wang Y. Zhang A. Wei X. Zhang Z. Bi X. Yuan X.. (2022b). Metagenomic next-generation sequencing identified psittacosis among poultry processing workers in Shandong Province, China. Inf. Med. 1, 135–139. 10.1016/j.imj.2022.06.001 38073879 PMC10699661

[B8] Zhou H. Xu L. Shi W. (2022). The human-infection potential of emerging tick-borne viruses is a global public health concern. Nat. Rev. Microbiol. 21, 215–217. 10.1038/s41579-022-00845-3 36526809

